# Environmental Detection and Potential Transmission of Equine Herpesviruses

**DOI:** 10.3390/pathogens10040423

**Published:** 2021-04-01

**Authors:** Anisha Dayaram, Peter A. Seeber, Alex D. Greenwood

**Affiliations:** 1 Department of Wildlife Diseases, Leibniz Institute for Zoo and Wildlife Research (IZW), 10315 Berlin, Germany; anisha.dayaram@charite.de (A.D.); seeber.pa@gmail.com (P.A.S.); 2 Institute of Neurophysiology, Charité—Universitätsmedizin Berlin, Charitéplatz 1, 10117 Berlin, Germany; 3 Limnological Institute, University of Konstanz, 78464 Konstanz, Germany; 4 Department of Veterinary Medicine, Freie Universität Berlin, 14163 Berlin, Germany

**Keywords:** EHV, viral transmission, equids, environmental

## Abstract

Equine herpesviruses (EHV) are a major health concern for domestic and wild equids and represent one of the most economically important disease agents of horses. Most known EHVs are transmitted directly between individuals as a result of direct exposure to exudates and aerosols. However, accumulating evidence suggests that environmental transmission may play a role including air, water, and fomites. Here, we reviewed studies on environmental stability and transmission of EHVs, which may influence viral dynamics and the use of environmental samples for monitoring EHV shedding.

## 1. Introduction

Equine herpesviruses (EHV) infect wild and domestic equine species worldwide and represent one of the most economically consequential infections of domesticated horses [1]. EHVs characterized to date belong to either the *gammaherpesvirinae* (EHV-2, -5, and -7) or *alphaherpesvirinae* (EHV-1, -3, -4, -6, -8, and -9) subfamily. Different EHV species present variable clinical manifestations with mild to severe symptoms including respiratory disease, abortion, coital exanthema, neonatal death, immunosuppression, and myeloencephalopathy [2]. Herpesviruses, including EHV, can replicate lytically, enabling transmission from host to host, or enter latency in which the host remains infected but viral shedding does not occur [3].

**Figure 1:**
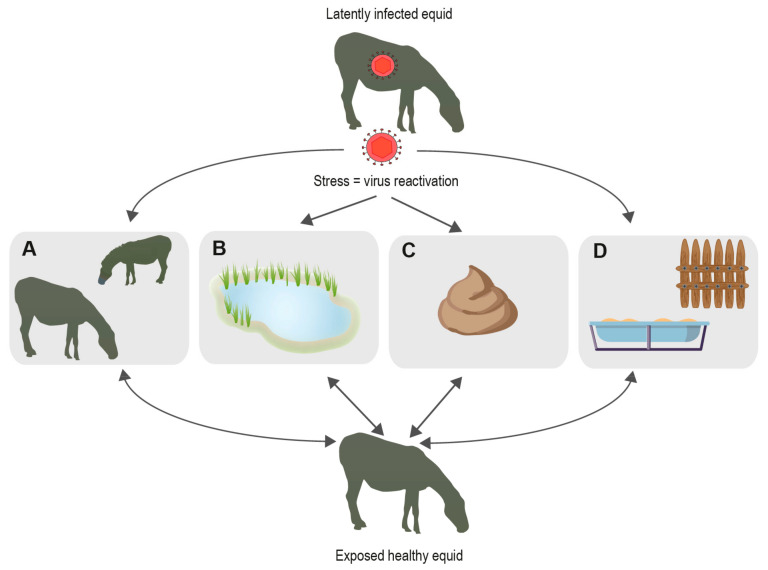
Known and possible EHV transmission pathways. When hosts are under stress, EHV is likely to become reactivated enabling hosts to shed virus and spread via different pathways; (**A**) transmission through direct contact with other animals; (**B**) indirect transmission through water; (**C**) indirect transmission via fecal matter; (**D**) indirect transmission by fomites.

During lytic replication, EHV transmission between hosts occurs frequently by direct contact with nasal mucus of infected animals. However, other sources of transmission include contact with EHV-1-positive aborted fetuses and direct transplacental infection from mother to foal often resulting in multigenerational disease transmission [4]. Equine semen has also been shown to transmit EHV-3 [5]. Equine embryo transfer incurs risk of EHV-1 transmission when the virus attaches to the cellular layer surrounding the embryo [6]. These risks can be largely controlled through testing measures. However, abiotic routes of transmission present additional risks of viral transmission. Risks include contact with contaminated fomites, aerosols, and fecal matter (Figure 1). Several studies have demonstrated potential transmission by indirect contact pathways to be possible. It remains unclear which role each of these pathways plays with regard to EHV transmission dynamics. However, there is increasing evidence suggesting environmental transmission plays a role [7].

The majority of research on EHVs has been carried out in veterinary settings, with histology, immunology, and pathology performed on domestic or captive wild equids displaying symptoms associated with EHV. However, recent studies have begun to examine stability and infectivity of EHV in the environment in an effort to explain among-species transmission of EHVs, frequently under conditions where direct transmission of virus can be excluded [7,8,9]. For example, interspecies transmission of EHVs between animals kept in different zoo enclosures has been reported [8,9]. This finding detailed that equid enclosures were physically distant from those of non-equids and frequently resulted in fatal outcomes for the non-definitive (non-equid) hosts, indicating that abiotic transmission may play a larger role in the transmission of EHV than anticipated [8,9]. There is a need to understand EHV transmission mediated by fomites, feces, air, and water that may be contributing to transmission among equids and to non-equids. This information will enable scientists to fully understand EHV transmission dynamics and develop effective control measures. Although there are vaccines available against the clinically relevant species EHV-1 and -4 for use in domestic horses [4], these still have many shortfalls. Current EHV-1/EHV-4 vaccines can prevent respiratory disease and reduce shedding but do not prevent the most severe form of EHV-1 disease, which is neurological disease, however, vaccinated individuals may still undergo subclinical virus reactivation and replication [10]. During this time, they may spread infectious material to naïve hosts. Furthermore, no vaccines against any other EHV species are currently available. Moreover, wild equids in captivity are infrequently vaccinated, which contributes to the urgency for monitoring environmental virus spreading. Treatment options for afflicted animals depend on clinical presentation and typically include antiviral medication and symptomatic therapy; however, it is important to note that equids may shed infectious material during clinical and subclinical (silent) infection and shortly thereafter. For this reason, physical isolation of virus shedders may help prevent virus transmission, which should include measures to prevent transmission via aerosols, fomites, or other environmental pathways.

In this review, we comprehensively summarize research on potential environmental transmission of EHVs from water, air, fomites, and feces, (Table 1) and how to detect and monitor EHVs from these substrates.

## 2. Transmission by Air

EHV transmission occurs primarily by direct contact between hosts, when EHV is shed in nasal discharge, which can develop into aerosol conveying infectious particles. There is evidence for aerosol transmission for both EHV-1 and -4 [16]. It is plausible that EHVs could either be spread via droplet transmission (particles > 5 µM) or by airborne transmission (particles < 5 µM) [11].

Herpesvirus-contaminated aerosols can travel many meters. Mars et al. 2000 showed that calves 4 m apart could become infected with bovine herpesvirus -1 (BHV-1) via aerosol transmission [20]. Both PCR of nasal swabs and antibodies specific to BHV-1 in serum were used to detect BHV-1 aerosol transmission between calves. The study also showed that some strains of BHV-1 transmit more effectively in aerosol form than others. In 2008, Pusterla and Mapes tested nebulized EHV-1 for airborne transmission [11]. They applied air sampling devices commonly used in pharmaceutical, medical, and industrial settings to investigate airborne pathogen dispersal. They demonstrated that nebulized EHV-1 could be detected by real-time PCR from air samples and environmental swabs following aerosolization of the virus. However, the study could not determine the maximum distance airborne EHV-1 particles travel through the air, which is thought to mainly occur by dispersal of respiratory secretion droplets containing EHV-1. The lack of precision was due to the many parameters including virus size, settling velocity, nature of the fluid containing virus, and environmental conditions such as temperature, airflow, and relative humidity that had to be accounted for [21]. To date, there are few reports of EHV-1 spreading naturally via aerosol droplets. During an incident when physically separated horses in an equine hospital became infected with EHV-1, it was speculated that transmission may have occurred through aerosolized droplets spreading from stall to stall [12]. However, as only one horse seroconverted, the results could not be distinguished from transmission via an alternative environmental route such as fomites. To control outbreaks, it remains imperative to understand the viral dynamics of aerosolized virus and the most effective methods to isolate EHV-infected animals in open stalls and enclosures.

## 3. Transmission by Fomites

A fomite can be described as an object that when contaminated with or exposed to a pathogen, can transfer the pathogen to a new host. The first studies examining the stability and infectivity of EHV-1 on different fomites were carried out in 1958 [22]. Different surfaces including white pine, galvanized iron, paper, Manila rope, horse tail hair, hessian fabric, and wheat straw were tested [22]. To test EHV-1 infectivity, equal titers of the virus were dried onto different fomite surfaces at temperatures of 20–27 °C. The results showed that the virus remained infectious for up to 7 days on surfaces including straw, glass, and galvanized iron. The virus also remained stable and infectious for up to 7 days on wood, paper, and Manila rope. On hessian fabric and horse hair, the virus remained infectious for 35–42 days after inoculation. The results from this study indicate that EHV stability on fomites is largely dependent on the surface it comes into contact with and that it can remain infectious up to 42 days after exposure. A recent study showed that EHV-1 can remain viable in the environment on materials such as leather, fabric, wood shavings, wheat straw, and polystyrene for at least 48 h [13]. It is unclear if animals can contract EHV infections from contaminated surfaces, which likely depends on the breed or species, dose of virus shed, exposure of virus to environmental factors such UV radiation and desiccation, and frequency of animal interaction with such surfaces.

An outbreak of EHV between separated North and South wings of a veterinary hospital is an example of how fomites may be responsible for some EHV-1 transmission. In this case, animals were physically separated and the infections were believed to be the result of exposure of hosts to contaminated fomites. This included equines being exposed to contaminated hands and clothing of personnel [12], suggesting that viruses are not only stable on fomites but that they may act as abiotic vectors of transmission.

A further study has shown that fomites may prove useful for non-invasive monitoring of EHV in both captive and wild equine species [15]. This study used a commercially available horse enrichment toy to examine EHV presence in captive Grévy’s, mountain, and plains zebra (*Equus grevyi*, *E. zebra,* and *E. quagga*). The toys were placed in outdoor enclosures for 7–24 h. Swabs were then taken from the surface of the toys and tested for EHV using a panherpes PCR screen. Four EHV species (EHV-1, EHV-7, wild ass herpesvirus, and zebra herpesvirus) were detected by PCR and subsequent sequencing. Host mitochondrial DNA was used to identify individual zebras that had interacted with the toy. The study showed that fomite-derived environmental DNA could be used to monitor the presence of pathogens such as EHV in the environment as well as identify individuals interacting with fomites.

## 4. Transmission through Feces

In contrast to herpesviruses of other taxa such as ruminants that may cause enteric diseases [23], relatively few studies have examined EHV shedding in feces since EHVs are assumed to be generally transmitted by nasal discharge (or venereal transmission as is typical in EHV-3 and may also occur in EHV-1) [24]. However, several EHVs exhibit neurotropism and have occasionally been found in the intestinal nervous tissue of horses [25,26,27]. Therefore, virus material may be discharged into the intestinal content during virus replication and systemic infection. Moreover, herpesvirus DNA in swallowed nasal exudate may endure the gut passage and therefore could be detectable in feces. Transmission by feces is speculated to occur through scent marking, which is frequent in wild and domestic equids and typically includes sniffing of feces or urine from conspecifics [28]. Furthermore, coprophagy may occur in young or starved equids [29,30], thus feces may be a potential source of infection.

The use of feces for EHV monitoring can facilitate sampling at larger scales and would be useful for screening active virus shedding in wild and domestic equids. In free-ranging and captive zebras, various EHV species were found to occur in feces, with EHV-5 and asinine herpesvirus 5 being most common [17]. However, EHV species detected in feces also included viruses that have been reported relatively rarely in previous studies (e.g., EHV-7, Wild Ass herpesvirus, and Equus zebra herpesvirus) [31]. Differences in tropism among viral species suggest that EHVs in feces may originate from both swallowed nasal exudate and from the enteric nervous system. In the case of nasal shedding, the host’s gut passage time must be considered, for example, EHVs stemming from swallowed nasal exudate would be expected to occur in feces only 1–2 days after the start of nasal virus shedding [32]. Compared to nasal swabbing, fecal EHV screening produces a higher rate of false-negative results [17], which, apart from presumably lower abundance of EHV in feces, may also be an effect of PCR-inhibiting substances common in feces (e.g., bile salts, plant secondary metabolites, and complex sugars) [17]. Thus, EHV screening of fecal samples may need to be optimized regarding the dilution of PCR template in order to obtain the optimal ratio of target DNA to PCR inhibitors. Several PCR protocols have been established to amplify herpesvirus DNA, and some of these methods are capable of detecting a very broad range of known and novel herpesviruses [33]. In this regard, nested PCR approaches are recommended when using fecal DNA as they are a more robust screening approach for complex substrates containing a high diversity of non-target DNA, such as feces [17]. However, viruses of wildlife may be insufficiently well characterized to be detected using domestic horse virus-based assays, which may limit obtaining results on the full spectrum of EHV diversity in a sample.

Other considerations need to be taken into account when using feces to monitor EHV shedding. Fecal samples should be collected fresh, as microbial metabolism may decrease the ability to detect viral DNA. The bolus surface typically contains more host intestinal cells than the interior, however, herpesviruses may originate from swallowed nasal exudate and may thus be well distributed within fecal matter; thus, subsamples should be mixed to optimize detection probability. DNA isolation kits for soil/feces typically do not include enzymatic digestion, however, host cells in feces or on swabs which contain herpesviruses may still be intact and thus require proteinase K digestion, which can typically be added to the protocol of non-tissue kits. In order to optimize detection efficiency, samples that likely contain considerable amounts of PCR inhibitors should be tested over a dilution gradient of the template DNA in order to determine the optimal ratio of target DNA and PCR inhibitors [17].

In contrast to other non-invasive methods, fecal sampling has the considerable advantage that it is possible to attribute samples to individual hosts. Moreover, for wild equids, fecal sampling may be the method of choice for noninvasive monitoring of EHV shedding when invasive procedures are impractical or impossible. In addition, fecal sampling allows for matching cumulative endocrine profiles with EHV viremia, as increased glucocorticoid levels can be associated with reactivation of latent herpesvirus infections [19,34]. Obtaining large numbers of invasive samples from wild equids is typically difficult, and therefore noninvasive methods may provide a tradeoff between false-negative results with much higher sample numbers and high effort associated with invasive sampling at lower sample numbers.

Regarding EHV-3, which is a venereal pathogen, urine rather than feces may be a useful substrate for tracing active viremia as this virus can be isolated from perineal and vaginal swabs of infected equids [35]. Urine from wild equids can be collected from hard surfaces using a syringe or from other substrates using cotton roll devices or by centrifugal filtration of soil samples; however, urine as a substrate for EHV screening has not been investigated.

## 5. Transmission by Water

For some viruses, including influenza, adenoviruses, hepatitis A virus, coxsackievirus, echovirus, reovirus and rotaviruses, stability and transmission in water is a common characteristic [36,37,38,39]. Many of these viruses have adapted to survive the harsh environment of the intestinal tract and by default are then able to survive in water [40]. However, the extent to which other viruses may utilize water is largely unknown. It remains to be shown if viruses have directly evolved to use water to facilitate transmission or if it is a consequence of another transmission route.

In the case of EHV-1, the virus was recently shown to remain stable and infectious in water under laboratory settings, indicating the potential to infect hosts by water [41]. The study demonstrated that EHV-1 is stable over a broad temperature (4–30 °C) and pH (pH 7–10) range. The results in water were similar to one of the first EHV-1 studies conducted, demonstrating the stability of the virus at up to 56 °C and in alkaline (pH 8–10) environments [22]. The study by Dayaram et al. further showed EHV-1 replication under a range of environmental conditions demonstrating the affinity of EHV-1 for water sediments [41]. It was proposed that the sediment could potentially protect the virus by preventing UV damage and thus potentially increasing the time the virus remains stable and infectious in water. This finding highlights that sediment, in addition to water, may warrant further investigation for their roles in EHV transmission. The ability of the virus to successfully replicate after long exposure to water in diverse aquatic conditions indicates that water and the sediment could potentially act as an indirect transmission vector between hosts.

To address how EHV-1 behaves in environmental water, a further study investigated EHV presence in environmental water and sediment. Samples were collected from countries known to have large equine populations including Mongolia, Tanzania, and Namibia [14]. Water and sediment samples were collected from a diverse range of environments including temperature (6.2–32.5 °C) and pH ranges (pH 5.5–8.1). The samples were screened for EHV resulting in the detection of EHV-1, -9, and -5. In addition, stability and infectivity were demonstrated through the direct culture of EHV-1 from water collected from sources in the South-East Gobi Desert, a location where several wild and domesticated equid species reside. Phylogenetic analysis of the recovered viral genomes showed that four isolates were similar to EHV-1 strain NMKT04 isolated in the UK. Other identified strains related to EHV-5 were highly divergent, and strains did not group according to geographic location, indicating global homogeneity of EHV viruses. This finding is similar to a report published by Garvey et al. 2019 that showed EHV genotypes are not geographically restricted [42].

Seasonal water restriction leads to animal aggregation, which can be associated with stress in zebras [14,17]. This rise in stress for animals may lead to increased viral shedding [19]. Increased shedding at limited water sources may increase the number of animals infected, which is an overall advantage to the virus. The laboratory-based EHV-1 stability experiments suggest that water shed into the waterholes may remain infectious for a substantial length of time (several weeks), increasing the risk of infection over an extended period of time. Many different species congregate at limited waterholes that could explain the observed cross-species transmission of EHV-1 and 9 in such ecosystems [43].

One of the factors that may drive the emergence of EHV through seasonal water is climate change [44]. As water sources become more restricted, it is likely that larger species assemblages congregating at the remaining sources will be at increased risk of exposure to EHV infections, and increased cross-species transmission frequency may be a consequence.

Animals in captivity do not face seasonal water restriction. However, their environment poses other opportunities for EHV-1 transmission by water. Shared water sources are a feature of many zoos and are often shared between enclosures of multiple animal species [8]. As different animal species in captivity rarely have direct contact, it is possible that shared water sources may be responsible for the increase in cross-species transmission events reported in captivity [8,45]. EHV-1 and -9 have been reported in captive Indian rhinoceros (*Rhinoceros unicornis*) [8], black bears (*Ursus americanus*) [9], Thomson’s gazelles (*Eudorcas thomsoni*) [46], guinea pigs (*Cavia porcellus* f. dom.) [9], and polar bears (*Ursus maritimus*) [45]. The infected animals often display typical symptoms associated with EHV including encephalitis and sever neurological disease; the severe nature of the symptoms has often resulted in infected animals being euthanized [47]. Phylogenetic analysis of EHV-1 and -9 sequences isolated from different species of zebra showed they were nearly identical to EHVs isolated from different equine species, highlighting the ability of both EHV-1 and -9 to cross species barriers. The transmission route of the viruses into these captive animals is still unknown. As the infected animals did not have direct contact with other animal species, transmission is assumed to have occurred through an indirect vector such as water.

## 6. Detection of Viral Shedding in the Wild on Fomites Using Swabs

In equids, nasal shedding is pre-dominant during viremia; thus, testing nasal swabs and invasively collected sample material such as blood is considered the most reliable method for monitoring EHV shedding. However, in the case of wild free living equids, collecting invasive samples generally requires physical restraint, which is typically associated with logistic and physical difficulties and may cause severe stress or risk to animal health [48,49].

As most EHVs are shed via the respiratory passage, active virus excretion can be traced by swabbing surfaces on which virus DNA was shed. Such surfaces include various objects in the immediate environment of equids on which nasal exudate was applied by direct contact or as an aerosol. In captive settings, swabbing of surfaces such as feed troughs and behavioral enrichment toys has been found to be an effective approach for monitoring acute EHV shedding in Grévy’s, mountain, and plains zebra [15,19]. Regarding wild equids in more natural environments, this approach may not be feasible due to lower encounter rates or lack of interaction with particular objects or lack of adequate surfaces to swab.

At present, very few studies on the effect of physicochemical factors on viability of EHVs in the environment are available [13,22,41]. Therefore, in order to prevent EHV spreading in captive or domestic equids, disinfection of surfaces may be recommendable under certain circumstances. However, the complexity of various environmental structures in domestic, captive, or natural settings may not permit efficient chemical or physical disinfection, thus allowing sufficient time after contamination by a virus-shedding animal, which may be equally important to allow for natural virus inactivation in the environment.

For swabbing dry surfaces, cotton swabs should be moistened using sterile water or other liquids such as sterile phosphate-buffered saline [15] so as to increase the amount of virus material adhering to the swab. It should be considered that DNA in environmental samples can be destroyed by UV light, which may affect detectability of EHV in sample material that has been exposed to sunlight; thus, swabs should be collected as soon as possible. Moreover, environmental surface swabs may be expected to contain considerable amounts of PCR inhibitors, thus DNA isolation kits for soil or feces, which typically include an inhibitor removal step, are recommended rather than extraction kits for mucosal swabs [17].

## 7. Conclusions

There is a growing body of evidence suggesting that different pathways may affect the transmission of EHV within and among species. Several EHV species are commonly found in the environment (e.g., EHV-1, EHV-5, -7, and asinine herpesvirus 5), whereas others such as EHV-4, -6, and -8 have not been detected in environmental sample material thus far. This is surprising for species such as EHV-4 that are highly prevalent with over 90% of domestic and wild equid populations infected [50,51] and with infectious virions being shed through nasal exudate. Detection in the environment likely depends on virus- and host-species-specific factors. EHV species that occur frequently in substrates such as nasal exudate or systemically in blood may be expected to be more likely to be shed directly into the environment, compared to EHVs that predominantly occur in nervous tissue. By contrast, species such as EHV-3, which is a strictly venereal pathogen, would not be expected to occur in samples that are not contaminated by genital mucosae (e.g., urine) and are thus not likely to be detected in other types of environmental samples.

Direct contact with infected animals and exposure to aerosols, infected respiratory secretions, placenta and uterine fluids, and fetal tissue from aborted fetuses remain the most common and direct pathways for EHV transmission between hosts; however, indirect pathways may play an important role in viral transmission in both captivity and nature. EHV transmission via fomites is of particular concern with the viruses remaining infectious on many different materials for extended lengths of time, which poses a particular risk for cross-species transmission in captive animal facilities where animals that would not be exposed to EHVs in their natural habitats may come into contact through an indirect pathway. Furthermore, studies are need to fully understand how pathogenic EHV-1 strains might transmit through aerosol droplets to infect equids in nearby enclosures. The equine industry would benefit from precise guidelines for distances in which equids can be safely housed without the risk of disease transmission. In addition, fecal matter now needs to be treated as a possible fomite for all EHV transmission until further studies are performed to show if fecal matter contains intact virions or just naked viral DNA. Better understanding of these different transmission pathways is of particular interest to captive animal facilities such as zoos, where exotic animals face high likelihoods of exposure and limited immunity to viruses such as EHVs. Zoo keepers and veterinarians working with equids need to be aware and understand how to isolate sick animals and prevent further spread of infection throughout facilities via indirect pathways, including fomites, aerosols, water, and feces. In natural settings, researchers need to better understand how global climate changes and destruction of habitat will increase direct and indirect contact rates among wildlife. Understanding all transmission pathways of EHV may allow for better prediction of inter and intraspecific viral transmission.

## Figures and Tables

**Table 1:** Non-invasively traced EHVs in different environmental sample types.

EHV Family	Synonym	Disease Manifestations	Air	Fomites	Water	Feces	Reference
*Alphaherpesvirinae*							
EHV-1	AsHV-3	respiratory disease, abortion, myeloencephalopathy	****•****	**•**	**•**	**•**	[1,11,12,13,14,15]
EHV-3		coital exanthema, respiratory disease					
EHV-4		respiratory disease	**•**				[16]
EHV-6	AsHV-1	mucosal lesions					
EHV-8	AsHV-3	respiratory disease, abortion, myeloencephalopathy					
EHV-9	Gazelle HV	respiratory disease, abortion, myeloencephalopathy					
*Gammaherpesvirinae*							
EHV-2		respiratory disease, conjunctivitis, pharyngeal ulceration		**•**		**•**	[17,18]
EHV-5		pulmonary fibrosis		**•**		**•**	[17,18]
EHV-7		abortion, fever, lethargy		**•**		**•**	[17,18,19]
Equus zebra HV		*uncertain*		**•**		**•**	[17,18,19]
Wildass HV		pneumonia				**•**	[18,19]
AsHV-4		respiratory disease, pneumonitis					
AsHV-5		*uncertain*				**•**	[17]
AsHV-6		*uncertain*					

AsHV refers to asinine herpesvirus. Reference numbers for the supporting literature are found in the reference list at the end of the manuscript. Filled circles represent viruses for which information on environmental detection exists in the peer reviewed literature.

## References

[1] Reed S.M., Toribio R.E. (2004). Equine herpesvirus 1 and 4. Vet. Clin. Equine Pract..

[2] Lunn D., Davis-Poynter N., Flaminio M., Horohov D., Osterrieder K., Pusterla N., Townsend H. (2009). Equine herpesvirus-1 consensus statement. J. Vet. Intern. Med..

[3] Van Maanen C. (2002). Equine herpesvirus 1 and 4 infections: An update. Vet. Q..

[4] Patel J., Heldens J. (2005). Equine herpesviruses 1 (EHV-1) and 4 (EHV-4)–epidemiology, disease and immunoprophylaxis: A brief review. Vet. J..

[5] Pascoe R. (1981). The effect of equine coital exanthema on the fertility of mares covered by stallions exhibiting the clinical disease. Aust. Vet. J..

[6] Hebia I., Fiéni F., Duchamp G., Destrumelle S., Pellerin J.-L., Zientara S., Vautherot J.-F., Bruyas J.-F. (2007). Potential risk of equine herpes virus 1 (EHV-1) transmission by equine embryo transfer. Theriogenology.

[7] Azab W., Dayaram A., Greenwood A.D., Osterrieder N. (2018). How host specific are herpesviruses? Lessons from herpesviruses infecting wild and endangered mammals. Annu. Rev. Virol..

[8] Abdelgawad A., Azab W., Damiani A.M., Baumgartner K., Will H., Osterrieder N., Greenwood A.D. (2014). Zebra-borne equine herpesvirus type 1 (EHV-1) infection in non-African captive mammals. Vet. Microbiol..

[9] Wohlsein P., Lehmbecker A., Spitzbarth I., Algermissen D., Baumgärtner W., Böer M., Kummrow M., Haas L., Grummer B. (2011). Fatal epizootic equine herpesvirus 1 infections in new and unnatural hosts. Vet. Microbiol..

[10] Foote C.E., Love D.N., Gilkerson J.R., Wellington J.E., Whalley J.M. (2006). EHV-1 and EHV-4 infection in vaccinated mares and their foals. Vet. Immunol. Immunopathol..

[11] Pusterla N., Mapes S. (2008). Evaluation of an Air Tester for the Sampling of Aerosolised Equine Herpesvirus Type 1. Vet. Rec..

[12] Goehring L., Landolt G., Morley P. (2010). Detection and management of an outbreak of equine herpesvirus type 1 infection and associated neurological disease in a veterinary teaching hospital. J. Vet. Intern. Med..

[13] Saklou N.T., Burgess B.A., Ashton L.V., Morley P.S., Goehring L.S. (2020). Environmental persistence of equid herpesvirus type-1. Equine Vet. J..

[14] Dayaram A., Seeber P., Courtiol A., Soilemetzidou S., Tsangaras K., Franz M., McEwen G., Azab W., Kaczensky P., Melzheimer J. (2021). Seasonal host and ecological drivers may promote restricted water as a viral vector. Sci. Total Environ..

[15] Seeber P.A., Soilemetzidou S.E., East M.L., Walzer C., Greenwood A.D. (2017). Equine behavioral enrichment toys as tools for non-invasive recovery of viral and host DNA. Zoo Biol..

[16] Ataseven V.S., Dağalp B.S., Başaran Z., Keskin S. (2010). Seroepidemiological studies of equine herpesviruses 1 (EHV-1) and 4 (EHV-4) infections in working horses from the eastern Turkey. Vet. Fak. Derg..

[17] Seeber P.A., Dayaram A., Sicks F., Osterrieder N., Franz M., Greenwood A.D. (2019). Noninvasive Detection of Equid Herpesviruses in Fecal Samples. Appl. Environ. Microbiol..

[18] Seeber P.A., McEwen G.K., Löber U., Förster D.W., East M.L., Melzheimer J., Greenwood A.D. (2019). Terrestrial mammal surveillance using hybridization capture of environmental DNA from African waterholes. Mol. Ecol. Resour..

[19] Seeber P.A., Quintard B., Sicks F., Dehnhard M., Greenwood A.D., Franz M. (2018). Environmental stressors may cause equine herpesvirus reactivation in captive Grévy’s zebras (*Equus grevyi*). PeerJ.

[20] Mars M., De Jong M., Van Maanen C., Hage J., Van Oirschot J. (2000). Airborne transmission of bovine herpesvirus 1 infections in calves under field conditions. Vet. Microbiol..

[21] Garner J.S. (1996). Guideline for isolation precautions in hospitals. Am. J. Infect. Control..

[22] Doll E.R., McCollum W.H., Bryans J.T., Crowe M.E. (1959). Effect of physical and chemical environment on the viability of equine rhinopneumonitis virus propagated in hamsters. Cornell Vet..

[23] Eugster A.K. (1979). Isolation of bovine herpesvirus III from diarrheic feces. Vet. Microbiol..

[24] Walter J., Balzer H.-J., Seeh C., Fey K., Bleul U., Osterrieder N. (2012). Venereal Shedding of Equid Herpesvirus-1 (EHV-1) in Naturally Infected Stallions. J. Vet. Intern. Med..

[25] Pavone S., Sforna M., Gialletti R., Prato S., Marenzoni M.L., Mandara M.T. (2013). Extensive Myenteric Ganglionitis in a Case of Equine Chronic Intestinal Pseudo-obstruction Associated with EHV-1 Infection. J. Comp. Pathol..

[26] Whitwell K.E., Blunden A.S. (1992). Pathological findings in horses dying during an outbreak of the paralytic form of Equid herpesvirus type 1 (EHV-1) infection. Equine Vet. J..

[27] Del Piero F., Wilkins P.A., Timoney P.J., Kadushin J., Vogelbacker H., Lee J.W., Berkowitz S.J., La Perle K.M.D. (2000). Fatal nonneurological EHV-1 infection in a yearling filly. Vet. Pathol..

[28] Pluháček J., Tučková V., King S.R. (2019). Overmarking behaviour of zebra males: No scent masking, but a group cohesion function across three species. Behav. Ecol. Sociobiol..

[29] Boyd L.E. (1988). Time budgets of adult Przewalski horses: Effects of sex, reproductive status and enclosure. Appl. Anim. Behav. Sci..

[30] Crowell-Davis S.L., Houpt K.A. (1985). Coprophagy by foals: Effect of age and possible functions. Equine Vet. J..

[31] Bell S.A., Pusterla N., Balasuriya U.B.R., Mapes S.M., Nyberg N.L., MacLachlan N.J. (2008). Isolation of a gammaherpesvirus similar to asinine herpesvirus-2 (AHV-2) from a mule and a survey of mules and donkeys for AHV-2 infection by real-time PCR. Vet. Microbiol..

[32] Steuer P., Südekum K., Müller D.W.H.H., Franz R., Kaandorp J., Clauss M., Hummel J. (2011). Is there an influence of body mass on digesta mean retention time in herbivores? A comparative study on ungulates. Comp. Biochem. Physiol. Part A Mol. Integr. Physiol..

[33] Vandevanter D.R., Warrener P., Bennett L., Schultz E.R., Coulter S., Garber R.L., Rose T.M. (1996). Detection and analysis of diverse herpesviral species by consensus primer PCR. J. Clin. Microbiol..

[34] Webster Marketon J.I., Glaser R. (2008). Stress hormones and immune function. Cell. Immunol..

[35] Vissani M.A., Perglione C.O., Zabal O., Alvarez G., Thiry E., Barrandeguy M., Parreño V. (2020). Topical Ganciclovir Reduces Viral Excretion in Mares With Equine Coital Exanthema. J. Equine Vet. Sci..

[36] Seitz S.R., Leon J.S., Schwab K.J., Lyon G.M., Dowd M., McDaniels M., Abdulhafid G., Fernandez M.L., Lindesmith L.C., Baric R.S. (2011). Norovirus infectivity in humans and persistence in water. Appl. Environ. Microbiol..

[37] Bae J., Schwab K.J. (2007). Evaluation of murine norovirus, feline calicivirus, poliovirus, and MS2 as surrogates for human norovirus persistence in surface and groundwater. Appl. Environ. Microbiol..

[38] Bofill-Mas S., Albinana-Gimenez N., Clemente-Casares P., Hundesa A., Rodriguez-Manzano J., Allard A., Calvo M., Girones R. (2006). Quantification and stability of human adenoviruses and polyomavirus JCPyV in wastewater matrices. Appl. Environ. Microbiol..

[39] Espinosa A.C., Mazari-Hiriart M., Espinosa R., Maruri-Avidal L., Méndez E., Arias C.F. (2008). Infectivity and genome persistence of rotavirus and astrovirus in groundwater and surface water. Water Res..

[40] Ewald P.W. (1991). Transmission modes and the evolution of virulence. Hum. Nat..

[41] Dayaram A., Franz M., Schattschneider A., Damiani A.M., Bischofberger S., Osterrieder N., Greenwood A.D. (2017). Long term stability and infectivity of herpesviruses in water. Sci. Rep..

[42] Garvey M., Lyons R., Hector R.D., Walsh C., Arkins S., Cullinane A. (2019). Molecular characterisation of equine herpesvirus 1 isolates from cases of abortion, respiratory and neurological disease in Ireland between 1990 and 2017. Pathogens.

[43] Abdelgawad A., Damiani A., Ho S.Y., Strauss G., Szentiks C.A., East M.L., Osterrieder N., Greenwood A.D. (2016). Zebra alphaherpesviruses (EHV-1 and EHV-9): Genetic diversity, latency and co-infections. Viruses.

[44] Stommel C., Hofer H., Grobbel M., East M.L. (2016). Large mammals in Ruaha National Park, Tanzania, dig for water when water stops flowing and water bacterial load increases. Mamm. Biol..

[45] Donovan T., Schrenzel M., Tucker T., Pessier A., Bicknese B., Busch M., Wise A., Maes R., Kiupel M., McKnight C. (2009). Meningoencephalitis in a polar bear caused by equine herpesvirus 9 (EHV-9). Vet. Pathol..

[46] Ghanem Y., Fukushi H., Ibrahim E., Ohya K., Yamaguchi T., Kennedy M. (2008). Molecular phylogeny of equine herpesvirus 1 isolates from onager, zebra and Thomson’s gazelle. Arch. Virol..

[47] Greenwood A.D., Tsangaras K., Ho S.Y., Szentiks C.A., Nikolin V.M., Ma G., Damiani A., East M.L., Lawrenz A., Hofer H. (2012). A potentially fatal mix of herpes in zoos. Curr. Biol..

[48] Arnemo J.M., Ahlqvist P., Andersen R., Berntsen F., Ericsson G., Odden J., Brunberg S., Segerström P., Swenson J.E. (2006). Risk of capture-related mortality in large free-ranging mammals: Experiences from Scandinavia. Wildl. Biol..

[49] Dickens M.J., Delehanty D.J., Romero M. (2010). Stress: An inevitable component of animal translocation. Biol. Conserv..

[50] Borchers K., Ebert M., Fetsch A., Hammond T., Sterner-Kock A. (2006). Prevalence of equine herpesvirus type 2 (EHV-2) DNA in ocular swabs and its cell tropism in equine conjunctiva. Vet. Microbiol..

[51] Barnard B., Paweska J.T. (1993). Prevalence of antibodies against some equine viruses in zebra (*Zebra burchelli*) in the Kruger National Park, 1991–1992. Onderstepoort J. Vet. Res..

